# James Taylor

**Published:** 1896-07

**Authors:** J. S. Cassidy

**Affiliations:** Covington, Ky.


### Obituary.

### JAMES TAYLOR, M. D., D. D. S.

BY J. S. CASSIDY, M. D., D. D. S., COVINGTON, KY.

Momorial Address, Fiftieth Anniversary of the Ohio College of Dental Surgery.

The recollection by those who knew Dr. James Taylor most intimately, of the charmingly honest qualities of his nature, has not been dimmed by the intervening years since he was in our midst; those qualities of heart and mind so evident that the stranger's confidence was won at once, and retained during increasing years of friendship.

Long before I had the pleasure of knowing him personally, I had heard him spoken of with love and affection by many of the oldest and best people of that time; a fact to which might be attributed largely some oftentimes expressed opinions of my own of the relatively high standing in the professional and social world of those necessarily itinerant dental practitinners contemporaneous with him.

It is, however, of Dr. Taylor as the originator, the Nestor of dental education in all this region of our country west of the Alleghanies, that I wish to speak, as the man to whom the excluded honor is due of establishing and maintaining the Ohio College of Dental Surgery, under difficulties of which many of us here can have no conception.

As he himself has said, "there were times when discouragement and failure seemed to be the inevitable consequences" of his unselfish efforts. But the spirit of wisdom was upon him, and he continued to labor in the cause, for a grand and noble purpose, until success was assured; in proof of which we are gathered here to-day to offer to his memory the meed of praise, our gratitude and our love.

After several years of service as dean of this college, he resigned his onerous charge to other hands, but still kept in touch with the classes by occasional lectures, and as a member of the board of trustees, and when in his comparatively old age circumstances over which he had no control disrupted the faculty, he assumed the task of changing chaos into order, and almost despair into the brightest hope; for without him at that time these happy changes in the condition of affairs would probably not have been accomplished.

'Twas then that we younger members of the faculty learned to know him well. His optimistic views in regard to the immediate future of the institution permeated its very atmosphere, and encouraged us, impelled us, to renewed exertion. He had said more than once that he hoped and expected to live to see the day when one hundred students would be in attendance—a statement that seemed to us almost preposterous, for in those days young men were not disposed to enter the profession through the doors of even the few dental colleges then existing; but he was wiser than we thought. for were he living now he would see enrolled more than double the number of his prophecy.

Truly the enthusiasm with which he labored overmastered the passage of time; and although the leaves in his chaplet were mellowed, aye, seared, by the years that had gone, there hovered around him, and abided with him always, the nimbus of eternal youth. Is it any wonder that his colaborers were imbued with some of the ardor of his own sanguine personality?

Another characteristic in his nature was his unvarying kindness to students: and while strict in his demands that they should do their duty during his course of lectures, the per cent. that he gave them in the final consultation by the faculty bore heavily in the scale on the side of mercy.

His abilities as a teacher did not depend alone upon his superior mental endowments; he had systematic method, as well as intellect, to aid him in imparting the knowledge with which his mind was so abundantly stored. He was not, in the narrow sense, dogmatic, for in his lectures he quoted copiously from other authorities; but the students were never left in doubt as to the correct practice in a given case, or the probable truth or falsity of any theory involved in the subject under consideration.

In his intercourse with his colleagues, officially or otherwise, he always manifested the kindly character of the Christian gentleman; considerate for the feelings of others, his gentle disposition won all hearts. Amiability, friendship and love were his instinctive possessions. He enjoyed a mild joke, and wit and humor, yet words never passed his lips, nor were stories told in his presence, that a woman might not hear.

What more can I say?

The dream of his life is realized; the edifice of dental education, of which he was one of the two architects, is nearly completed. May peace be with him! The ages yet to come will produce many noble men, none, however, nobler than he.

Thou shalt continue to garner thy sheaves, O Death! but few will be the germs of pre-eminent manhood in thy harvest of golden grain that will excel, in all that the word implies, the honored subject of this memorial.—*Cosmos*.

---

